# From Bench to Bedside: Reframing the *CXCL13*/*CXCR5* Axis as a Non-Redundant Organizer of Protective Pulmonary Immunity in COVID-19

**Published:** 2026-07-22

**Authors:** Lbachir BenMohamed

**Affiliations:** 1 Laboratory of Cellular and Molecular Immunology, Gavin Herbert Eye Institute, University of California, Irvine, School of Medicine, Irvine, California 92697;; 2 Institute for Immunology, University of California, Irvine, School of Medicine, Irvine, California 92697;; 3 TechImmune, Inc, Irvine, California 92660; USA.

## DESCRIPTION

### Overview: From mouse to patient:

A central question in COVID-19 management is why some patients develop lifethreatening disease while others recover despite comparable viral burdens. Growing evidence increasingly points to the quality of the early pulmonary immune response. Using a *CXCL13*-deficient transgenic mouse model (*CXCL13*^−^/^−^K18-hACE2) infected with the SARS-CoV-2 Delta variant, we recently discovered that loss of a single homeostatic chemokine, *CXCL13*, produced markedly worse outcomes, higher lung viral loads, severe histopathological injury, accelerated weight loss, and increased mortality, mechanistically tied to depletion of lung-resident *CXCR5*+*CD19*+ B-cells, *CXCR5*+*CD4*+ follicular T-helper cells, and *IFN-γ+TNF*-α+GzmB+Ki67+ effector Th1 cells, alongside reduced Spike-specific IgG1 and IgG2b titers [1]. These data reframe *CXCL13* from a potentially harmful inflammatory mediator into a non-redundant organizer of protective antiviral immunity within the lung, with direct translational implications for how clinicians interpret immune biomarkers, counsel patients on vaccination, and consider emerging immunotherapeutic strategies.

### Resolving a longstanding clinical paradox:

Clinicians and researchers have faced conflicting evidence regarding *CXCL13* [2–4]. Elevated serum *CXCL13* has been reported as a predictor of disease severity and mortality in hospitalized COVID-19 patients, yet early *CXCL13* expression in the lung mucosa has been associated with survival in severe cases, and its absence has been observed in patients who progress to fatal disease. Our data offer a biologically coherent resolution. Locally expressed CXCL13 within the lungs, produced early in infection by resident B-cells and T-cells, orchestrates ectopic germinal center formation and promotes the differentiation of *CXCR5*+ lymphocytes capable of viral clearance [1]. The elevated serum *CXCL13* detected in severe and fatal cases likely reflects a consequence of overwhelming lung damage and systemic immune dysregulation, not a cause. Clinicians should therefore resist the temptation to interpret circulating *CXCL13* elevation as a target to suppress; doing so could inadvertently disable a protective mucosal circuit at the precise moment it is most needed.

### Clinical implication 1: CXCL13 as a contextual biomarker:

The distinction between local mucosal *CXCL13* signaling and systemic serum *CXCL13* elevation carries important prognostic implications. Rather than treating any detectable elevation as uniformly ominous, clinicians should consider the clinical context in which it arises. An early rise in *CXCL13*, particularly when accompanied by evidence of robust B and T-cell activity, rising lymphocyte counts, appropriate antibody responses, and improving clinical trajectory, may signal effective immune mobilization rather than imminent deterioration. Conversely, persistently and markedly elevated *CXCL13* in the context of worsening oxygenation, progressive radiographic infiltrates, rising inflammatory markers, and T-cell exhaustion may reflect pathological immune activation and fibrogenic signaling. Future prospective studies should define threshold values and kinetic patterns of serum *CXCL13* that discriminate protective early responses from maladaptive late-stage inflammation. Until such data are available, *CXCL13* should be interpreted in the clinical context, not in isolation.

### Clinical implication 2: Implications for vaccination strategy:

Our findings have direct relevance to vaccine design and clinical deployment. *CXCL13* production in the lung appeared as early as day 2 post-infection in our model, produced by lung-resident B-cells and T-cells in a virus-nonspecific manner. This early chemokine release is critical for organizing the *CXCR5*+ cellular machinery needed for effective antigen-specific humoral responses, and intramuscular injection alone may be insufficient to prime this local pulmonary *CXCL13*/*CXCR5* circuit, an inference supported by published data in parallel systems [5–9]. For clinicians counseling patients about booster vaccination, particularly immunocompromised individuals or those with impaired mucosal immune responses, our findings provide a mechanistic rationale for mucosal or intranasal vaccine platforms that may more effectively engage the *CXCL13*/*CXCR5* axis at the site of infection [1].

### Clinical implication 3: Toward CXCL13-based immunotherapy:

Perhaps the most translatable insight from our work is the potential for *CXCL13* pathway agonism as a therapeutic strategy. That *CXCL13* deficiency was not compensated by upregulation of other mucosal chemokines, including *CXCL14*, *CXCL17*, *CCL25*, and *CCL28*, underscores the non-redundant role of this axis and suggests that augmenting rather than suppressing *CXCL13*/*CXCR5* signaling early in infection could accelerate viral clearance and reduce progression to severe disease. Candidate strategies include inhaled *CXCL13* as an adjuvant, intranasal delivery platforms that stimulate local *CXCL13* production, or small-molecule *CXCR5* agonists. Importantly, our data do not support *CXCL13* inhibition during acute COVID-19, as *CXCL13*-deficient mice experienced dramatically worse outcomes. Broad immunosuppressive regimens that may inadvertently impair the *CXCL13*/*CXCR5* axis should therefore be monitored carefully in early disease, where preserving this pathway may be protective.

### Limitations and the road ahead:

Several caveats temper immediate translation. The constitutive *CXCL13* deficiency of our model may not replicate the transient insufficiency seen in human patients, and our focus on young healthy mice limits applicability to older adults with comorbidities. Extrapulmonary organ involvement was not examined, and whether these findings extend to Long COVID, where T-cell exhaustion and persistent inflammation driven by viral persistence across multiple organs are well-documented, remains to be determined. Conditional, cell-type-specific knockout models and aged animals with metabolic comorbidities will be essential to complete the translational bridge to clinical practice.

## CONCLUSION

### Conclusion: A chemokine worth watching:

The *CXCL13*/*CXCR5* chemokine axis is not merely a passive bystander in COVID-19 immunity; it is a critical organizer of protective lung-resident B-cells and T-cells. Our preclinical data compel a reappraisal of how clinicians and investigators interpret *CXCL13* measurements, design vaccination strategies, and conceptualize immunotherapies for respiratory viral infections. As the field moves toward precision immunology in infectious disease, *CXCL13* belongs on the short list of immune mediators whose tissue context, temporal kinetics, and cellular sources must be defined rather than merely measured. The bedside payoff, better prognostication, more targeted vaccination, and immuneaugmenting therapies are within reach.
